# Shape-Based Tracking Allows Functional Discrimination of Two Immune Cell Subsets Expressing the Same Fluorescent Tag in Mouse Lung Explant

**DOI:** 10.1371/journal.pone.0039831

**Published:** 2012-06-22

**Authors:** Daniel Fiole, Cédric Touvrey, Anne Quesnel-Hellmann, Julien Douady, Jean-Nicolas Tournier

**Affiliations:** 1 Unité Interactions Hôte-Agents Pathogènes, Département de Microbiologie, Institut de Recherche Biomédicale des Armées, La Tronche, France; 2 Laboratoire Interdisciplinaire de Physique UMR 5588, Univ. Grenoble 1/CNRS, Grenoble, France; 3 Ecole du Val-de-Grâce, Paris, France; Ulm University, Germany

## Abstract

Dendritic Cells (DC) represent a key lung immune cell population, which play a critical role in the antigen presenting process and initiation of the adaptive immune response. The study of DCs has largely benefited from the joint development of fluorescence microscopy and knock-in technology, leading to several mouse strains with constitutively labeled DC subsets. However, in the lung most transgenic mice do express fluorescent protein not only in DCs, but also in closely related cell lineages such as monocytes and macrophages. As an example, in the lungs of CX_3_CR1^+/gfp^ mice the green fluorescent protein is expressed mostly by both CD11b conventional DCs and resident monocytes. Despite this non-specific staining, we show that a shape criterion can discriminate these two particular subsets. Implemented in a cell tracking code, this quantified criterion allows us to analyze the specific behavior of DCs under inflammatory conditions mediated by lipopolysaccharide on lung explants. Compared to monocytes, we show that DCs move slower and are more confined, while both populations do not have any chemotactism-associated movement. We could generalize from these results that DCs can be automatically discriminated from other round-shaped cells expressing the same fluorescent protein in various lung inflammation models.

## Introduction

The lung immune system is very efficient: constantly exposed to pathogens and pollutants, the lower respiratory airways are nevertheless maintained sterile, while inflammation is kept at the lowest level [1]. This *tour de force* is a result of strong evolutionary constraints to maintain the delicate architecture of alveoli intact and functional, allowing gas exchange at the alveolar-capillary interface. The lung immune system is then formed by individual cells dispersed along the surface of the respiratory tract [2]. The dynamics of this system have been approached only recently at the microscopic level by imaging technologies, mainly because the lung movements *in vivo* or the drift *ex vivo* do not accommodate an easy microscopic analysis [3].

Among the most important immune cells in the lungs are monocytes, alveolar macrophages and dendritic cells (DCs) [1]. Structurally, macrophages are mostly residing on the external side of the alveoli, while DCs lie in the interstitium [4]. Both alveolar macrophages and DCs are resident cells. In contrast, monocytes are mainly patrolling cells, forming in the case of infection an on-site, ready to use, and rapidly mobilizable subset. They are also known as precursors of macrophages and DCs in mouse lung [5]. To make the picture more accurate, DCs are not a unique population. Classically DCs are categorized as plasmacytoid DCs and conventional DCs [6]. In the lung at least two functionally distinct subsets of conventional DCs have been described, expressing either the integrins CD11b or CD103 [7], [8]. Most CD11b+ DCs are found in the submucosae, while CD103+ DCs are intraepithelial. Functionally, CD103+ are related to CD8α+ DCs and specialize in capturing apoptotic cells as well as activating CD8 T cells [9], [10]. CD11b DCs are prone to activate CD4 T cells and produce a wide array of chemokines [11], [12]. The CD11b subset will require a special attention here, because a majority of them express CX_3_CR1 [13]. As a result, transgenic CX_3_CR1^+/gfp^ mice form a good model for imaging a major DC population in the lung [14]. Interestingly, initial description of the CX_3_CR1^+/gfp^ mouse strain clearly showed that the enhanced Green Fluorescent Protein (EGFP) is expressed in different organs in various myeloid cells such as Küpfer cells in the liver, and glial cells in the brain. Within lymphoid organs EGFP is expressed in different cell subtypes including DCs, monocytes and NK cells [15]. In the lung, two main subsets including resident Gr-1^low^ monocytes [5], [16], [17] and CD11b^+^ DCs express EGFP in CX_3_CR1^+/gfp^ mice [18]. Using this strain for imaging studies does not allow a systematic discrimination of these two cell populations. So far, ex vivo analysis of DCs subsets by two-photon microscopy have been performed using MHCII-EGFP [19], and CD11c-YFP [20], [21] knock-in mouse strains, in trachea and lung explant, respectively. However the same issue about the discrimination of macrophages and DCs arises with these two models, due to their shared marker expression in the lung.

**Figure 1:**
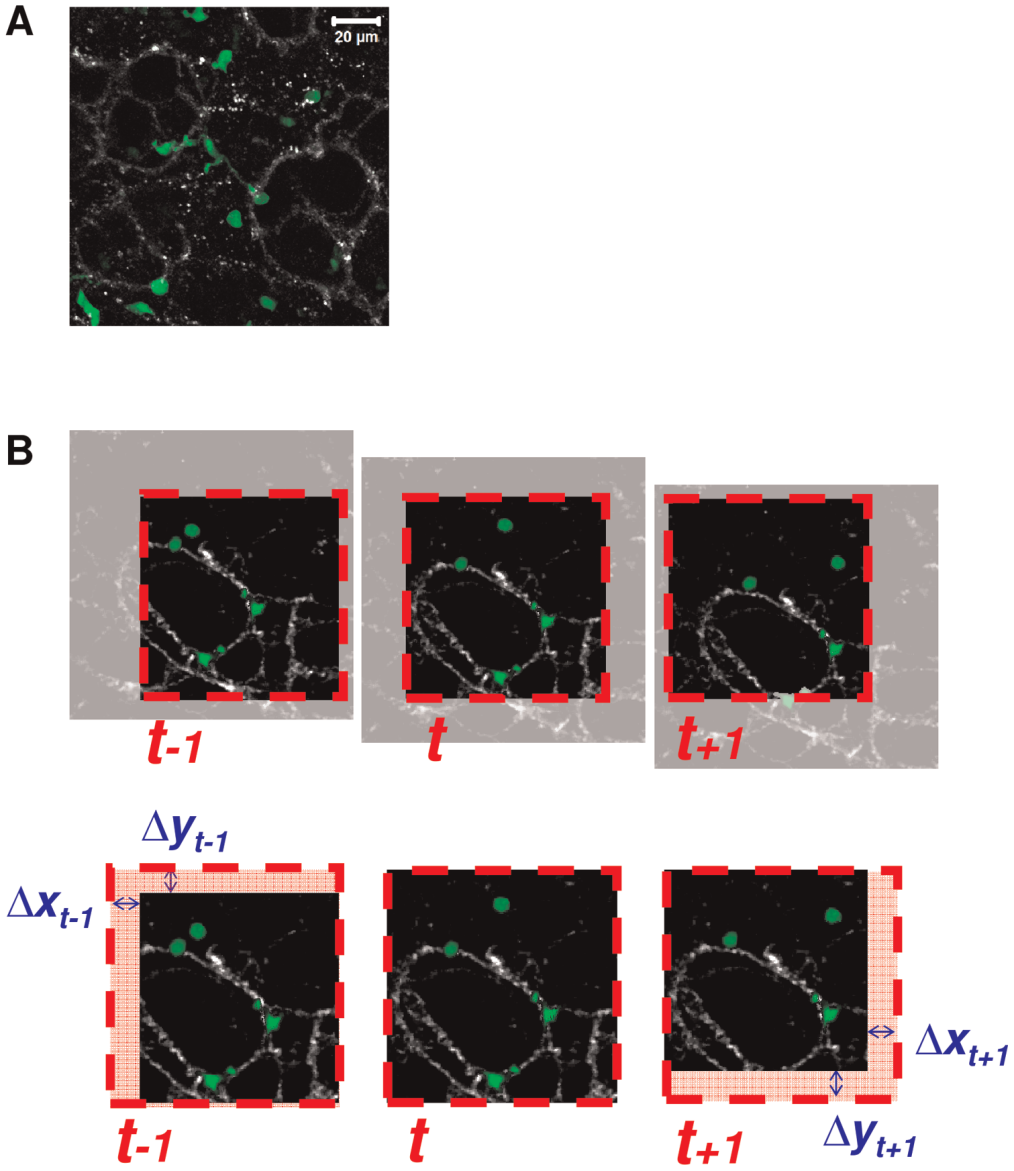
Schematic representation of the drift correction strategy. A: Maximum intensity projection of a lung slice z-stack. Pulmonary CX_3_CR1-GFP cells (green) and alveolar collagen mesh detected by collection of SHG signal (gray). Two-photon excitation wavelength  = 896 nm. B: Dashed red squares show the optic field imaged by the microscope (CX_3_CR1-GFP in green and SHG in grey). The realignment phase consists in calculating the tissue drift using the maximization of SHG signal cross-correlation.

The aim of the present study is to show how to overcome the non-discrimination of different subsets sharing the same fluorescent tag expression in dynamic studies. Here, we demonstrate the feasibility of an automated discrimination of two main CX_3_CR1-positive cell populations using a criterion based on the cell shape: the roundness. In order to separate Round-shaped cells (RSCs) and Dendritic-shaped Cells (DSCs), we suggest to introduce two novel coefficients: the Instantaneous Roundness Coefficient (IRC) measured in each frame and the Mean Roundness Coefficient (MRC) calculated as the mean of the IRC on the total tracking time for each cells. Using this strategy implemented in a cell tracking code, we show that different behaviour can be observed between the Round-shaped Cell (RSCs) and Dendritic-shaped Cell (DSCs) subsets. This novel approach may be generalized to other transgenic animal strains (e.g. MHCII-EGFP and CD11c-YFP knock in mice). This could lead to a better understanding of DC behaviour and a better analysis of the lung immune system during infection.

**Figure 2:**
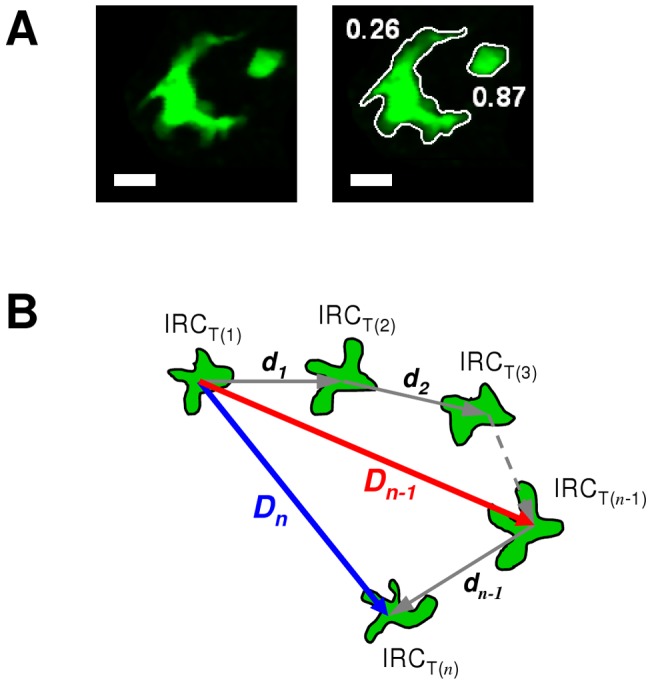
Parameters used for individual cell analysis. A: Edge detection of two CX_3_CR1+ pulmonary cells and their roundness coefficient. Scale bar  = 10 µm. B: The relevant parameters used in this work are: i) the Mean Roundness Coefficient (MRC), calculated for each cell by meaning Instantaneous Roundness Coefficient (IRC) at each consecutive observable time; ii) the Maximal Distance (MD) of a cell (red arrow) is the longest distance covered from the first position; iii) the Meandering Index (MI) is the final distance from the first position *D_n_* divided by the total length covered.

## Methods

### Ethics Statement

All experimental procedures were performed in accordance with the French Government guidelines for the care and use of laboratory animals and were approved by the *Institut de Recherche Biomédicale des Armées* ethics committee (approval number: 2010/28.0).

### Animal Care Guidelines

CX_3_CR1^+/gfp^ mice (further referred as CX_3_CR1 mice) were maintained under specific pathogen-free conditions at the *Plate-Forme de Haute Technologie Animale* (Institut Jean Roget, La Tronche, France). Mice were kept under anesthesia during all manipulations using ketamine-xylazine and all efforts were made to minimize suffering. The working solution is composed of 20% Ketamine 1000 (Vibrac) and 5% Rompun (Bayer Healthcare) diluted in PBS and was injected by intraperitoneal route (i.p.). To mimic the effects of Gram-negative bacteria lung infection, 120 µg of lipopolysaccharide (LPS) from *Escherichia coli* (LPS-EB ultrapure, InvivoGen) in a total volume of 55 µL diluted in PBS were delivered by intratracheal route (i.t.). Control mice received an equivalent volume of PBS.

**Figure 3:**
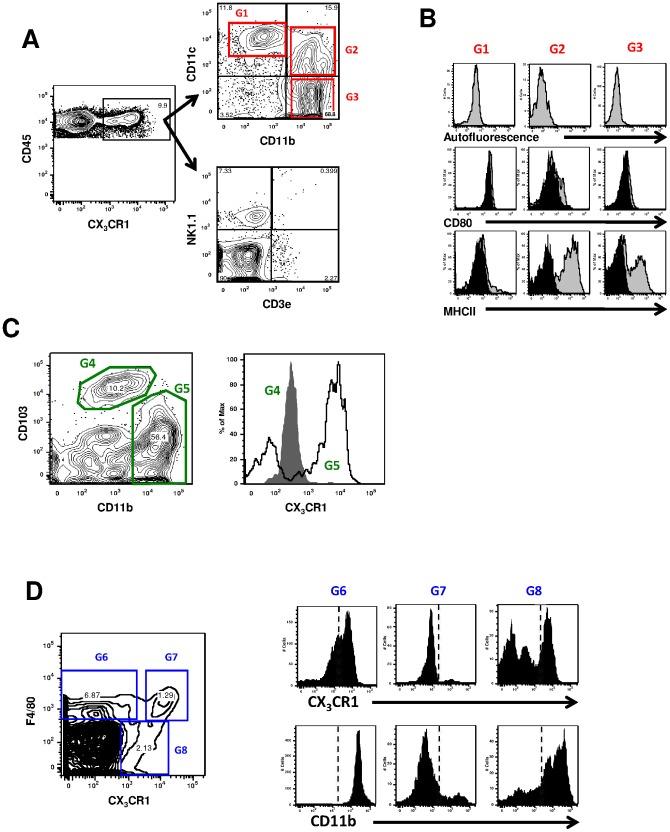
Phenotype of CX_3_CR1-GFP cell subsets in the lung. A: Total lung cells of CX_3_CR1^+/gfp^ mice were gated on CX_3_CR1 and analyzed for NK1.1, CD3e, CD11c, and CD11b expressions. B: Autofluorescence, CD80 and MHCII expressions on gate G1, G2 and G3 of panel A. Black histogram, isotype control; grey histogram, positive staining. C: Total lung cells were pre-gated on CD11c+ low autofluorecent cells and analyzed for the expression of CD11b and CD103. The expression of CX_3_CR1 is shown on the left panel for gate G4 (CD11b^−^CD103^+^ DCs, grey histogram) and for gate G5 (CD11b^+^CD103^−^ DCs, black line). D: Total cells were pre-gated on CD45 cells and analyzed for the expression of F4/80 and CD11c. Expression of CX_3_CR1 and CD11b is shown on the left panels for gate G6 (CD11c^low^F4/80^high^), G7 (CD11c^high^F4/80^high^) and G8 (CD11c^high^F4/80^low^).Data from flow cytometry, performed on one CX_3_CR1^+/gfp^ mouse lung harvested 30 minutes after intratracheal PBS injection. Data are representative of two distinct experiments.

**Figure 4:**
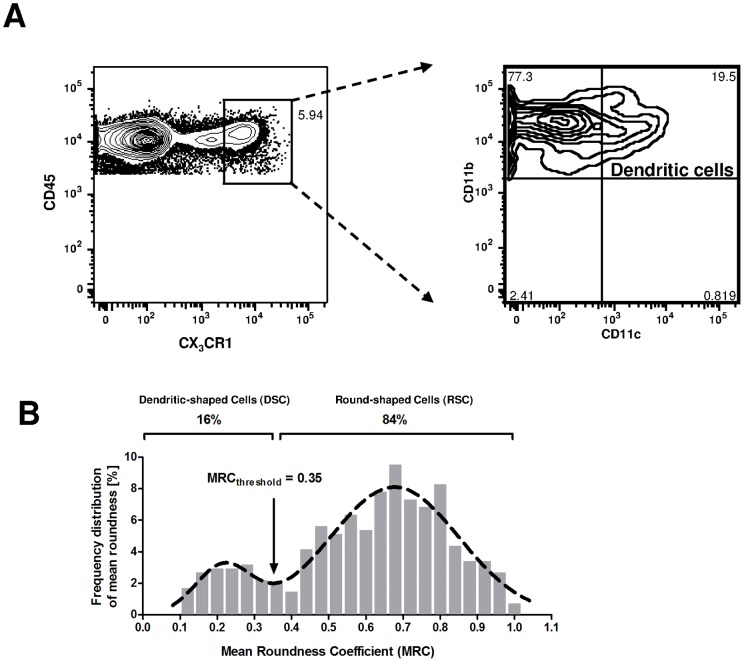
Discrimination of two CX_3_CR1-GFP cell subsets using flow cytometry or roundness. A: Expression of CX_3_CR1 *vs* CD45 receptors in pulmonary cells and CD11c *vs* CD11b by CX_3_CR1^high^ cells. Data from flow cytometry, performed on one CX_3_CR1^+/gfp^ mouse lung harvested 30 minutes after intratracheal PBS injection. Data representative of two distinct experiments. B: Frequency distribution of Mean Roundness Coefficients (MRC) over 1h of CX_3_CR1+ pulmonary cells in lung slices harvested from 6 mice, 1h30 (3 mice) and 5h (3 mice) after PBS injection. N = 406. Cells are followed for a mean time of 32 minutes, corresponding to 16 frames. Dashed line: sum of two Gaussian fitting the histogram. R^2^ = 0.89.

### Sample Preparation for microscopy

Mice were euthanized either 30 minutes (‘early stage’ group) or 4 hours (‘late stage’ group) post administration of PBS or LPS. Left lobes of lung explants were cut in the middle with a vibratome (Leica). The bottom of lung lobes was carefully glued on a Petri dish filled up with phenol-red free RPMI medium (RPMI 1640, PAN Biotech GmBH) at 37°C. Medium was refreshed every hour. Explants were kept for one hour at 37°C in a 5% CO_2_ environment before imaging and kept at 37°C during the whole experiment. This phase was aimed to stabilize the explant by emptying out most of the air from the alveoli.

### Flow Cytometry

Lungs were harvested after mouse euthanasia, mechanically disrupted using gentleMACS™ Dissociator (Miltenyi Biotec) according to manufacturer instructions, enzymatically digested with 1 mg/mL Collagenase I (Worthington) for 30 min at 37°C in 50U/mL DNase I (Sigma)-containing DMEM. Then, the solution was filtered with 70 μm cell strainers (Becton Dickinson) to obtain single-cell suspensions.

**Figure 5:**
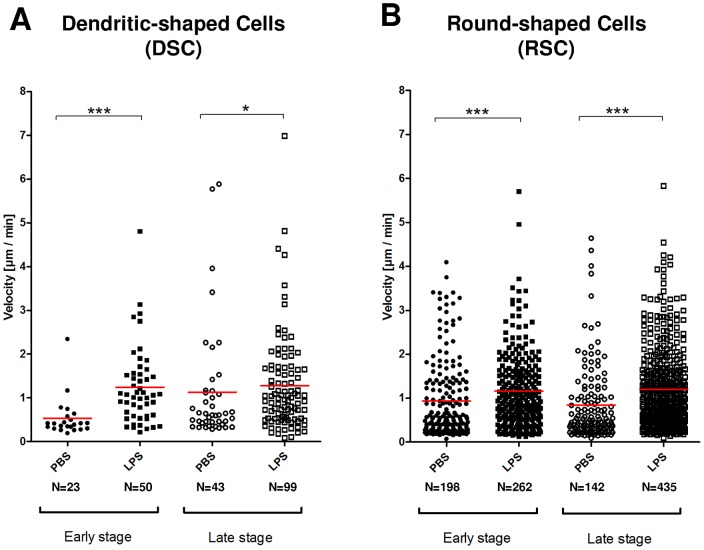
Velocity of CX_3_CR1-GFP positive pulmonary Dendritic-shaped and Round-shaped cells. A: Dendritic-shaped cells and B: Round-shaped cells at an early stage (average values from 1h30 to 2h30 post injection, closed symbols) and a late stage (average values from 5h to 6h post injection, open symbols) after injection of PBS (rounds) or LPS (squares). Three mice in each group, one symbol by cell. * for p<0.05; ** for p<0.01; *** for p<0.0001; ns for not significant.

Inhibition of nonantigen-specific binding of immunoglobulins to Fc receptors was performed using a rat antimouse CD16/CD32 antibody (2.4G2 BD Biosciences). Cells were subsequently stained for 30 min at 4°C with the following monoclonal antibodies: Alexa Fluor 700 conjugated anti-CD45 (30-F11; Biolegend), PE-Cy7 conjugated anti-CD11b (M1/70; eBioscience), APC conjugated anti-CD11c (HL3; BD Biosciences), PE conjugated anti-NK1.1 (PK136; BD Biosciences), PercPCy5.5 anti-CD3e (145-2C11; eBioscience), Alexa Fluor 700 conjugated anti-MHC II (MC-114-15.2; eBioscience), PercPCy5.5 anti-F4/80 (BM8; Biolegend), PE conjugated anti-CD80 (16-10A1; BD Biosciences), PE conjugated anti-CD103 (2E7; eBioscience).

**Figure 6:**
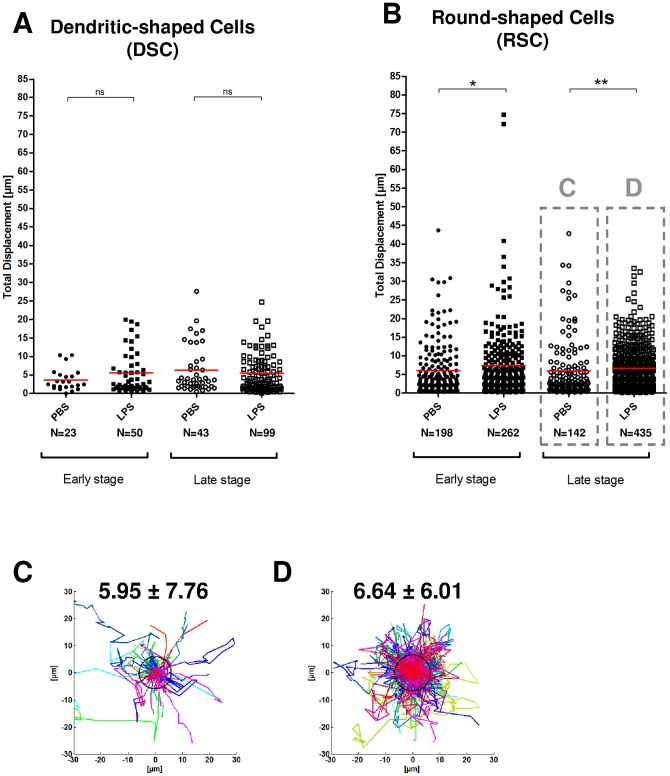
Maximal Distance of CX_3_CR1-GFP positive pulmonary Dendritic-shaped and Round-shaped cells. A: Dendritic-shaped cells and B: Round-shaped cells at an early stage (average values from 1h30 to 2h30 post injection, closed symbols) and a late stage (average values from 5h to 6h post injection, open symbols) after injection of PBS (rounds) or LPS (squares). Three mice in each group, one symbol by cell. C, D: Overlay of Round-shaped cell tracks after late PBS (C) and LPS injection (D), after aligning their first coordinates. One color by track. Values of black circle radii in µm, equal to average cell Maximal Distance, are indicated ± standard deviation. * for p<0.05; ** for p<0.01; *** for p<0.0001; ns for not significant.

Dead cells were excluded by staining for 30 min at 4°C with Blue LIVE/DEAD® Fixable Dead Cell Stains (Invitrogen) following the manufacturer instructions.

Cells were then fixed with Cellfix (BD Biosciences). Cell acquisition was directly performed with an LSR-II machine using FACSDiva software (BD Biosciences) and the data were analysed with FlowJo software (TreeStar). Cell doublets were excluded using FSC-A and FSC-H.

### Microscopy Setup

Both second-harmonic generation (SHG) and two-photon excitation fluorescence (TPEF) imaging were performed on a Zeiss LSM 710 microscope equipped with a W Plan-Apochromat 20× NA 1.0 DIC M27 75mm water immersion objective (Zeiss). Two-photon excitation was produced at 896 nm by a femtosecond Ti: Sa laser (Chameleon Ultra, Coherent). SHG and EGFP signals were both epidetected by two dedicated non-descanned detector, one is coupled with a 500–550 nm band-pass system for EGFP and the other with a 448 nm ±20 nm band-pass filter for SHG. Z-stacks were acquired every two minutes during one hour. Images size was 512 by 512 pixels, corresponding to a field of view of 280 by 280 µm.

### Cell Tracking

Data processing was performed under Matlab using a multiple particle tracking code by Blair and Dufresne (available on http://physics.georgetown.edu/matlab/).

### Statistical analysis

For the four experimental conditions (mixing early/late stage and PBS/LPS delivery), results from three mice were pooled. Comparisons between groups were performed using Mann-Whitney test, using GraphPad Prism Software (GraphPad Software, Inc.).

## Results

### Drift Correction

Sample drift due to the presence of air in the lung is a major issue [22], as shown by the common use of trachea, a more rigid tissue used as an alternative to lung tissues. In other organs, the drift may be negligible compared to cell speed, but in the lung it has the same range. We used the collagen SHG signal as a spatial reference to correct this drift. The realignment of 2D-projected frames was based on maximization of the spatial cross-correlation function of SHG signal using a custom made Matlab (MathWorks) code (Figure 1A and 1B).

### Cell tracking strategy

The Matlab code was implemented with an edge detection custom-made routine aimed to determine the roundness coefficient of each cell. Instantaneous Roundness Coefficient (IRC) is defined as follow:
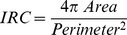



The IRC indicates the index of circularity of any object: from 0 (line-shaped) to 1 (round-shaped) (Figure 2A). Because the IRC of a particular cell changes along its displacement in pulmonary tissue all over the experiment, we suggested to use the Mean Roundness Coefficient (MRC) attributed to the cell according to the following formula (Figure 2B) where *n* is the number of consecutive frames on which the cell is observed:
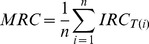



The Meandering Index (MI) yields information about the directionality of the cell movement [22] and is defined as the following (Figure 2B):
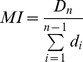



This parameter being inappropriate to fully characterize a random walk path [23], we also used the Maximal Distance (MD), which is for a cell the highest distance reached from its first position, to improve the cell confinement description (Figure 2B):
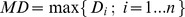



### Discrimination of lung CX_3_CR1^+^ Round-shaped Cells and Dendritic-shaped Cells subpopulations

First, we assessed the different lung phagocyte subpopulations by flow cytometry analysis (Figure 3). Initially, we demonstrated that CX_3_CR1 is expressed at high level within a subset of NK1.1 cells, CD3e cells, CD11b^+^CD11c^−^ monocytes, and CD11c^+^CD11b^+^ DCs (Figure 3A). However, this expression was not observed on CD11c^high^CD11b^−^ macrophages (Figure 3B, gate G1), as the CX_3_CR1 intermediate levels of fluorescence correspond to autofluorescence. As two main subsets of DCs have been described in the lung, we also demonstrated that CX_3_CR1 is expressed in a subset of CD11b^+^CD103^−^-DCs, but not in the CD11b^−^CD103^+^ population (Figure 3C). Utilizing F4/80 in conjunction with CD11c we were able to confirm that DC defined as CD11c^+^F4/80^−^ cells express various level of CX3CR1 as previously shown (Figure 3D) [24]. F4/80^+^CD11c^−^ myeloid cells expressed low level of CX3CR1 while CD11c^high^F4/80^high^ alveolar macrophages did not expressed CX3CR1 as expected. In the microscopy analysis, we focused on the CX_3_CR1^high^ population, excluding CX_3_CR1^int^ population that contains macrophages. In accordance with previous studies, CX_3_CR1^+^ population represents about 6% of total CD45^+^ lung cells [13] (Figure 4A). Using classical double CD11b/CD11c staining we could differentiate in CX_3_CR1-positive cells, two subpopulations expressing either CD11b only (mostly monocytes, about 77%), or CD11b and CD11c (DCs, about 19.5%).

To validate our imaging analysis approach, we first looked at the representation of distribution frequency of MRC at homeostasis (Figure 4B). Interestingly, we could observe two populations. We applied a double Gaussian fit on the distribution, which exhibited a local minimum, splitting the total population into two subpopulations for a MRC threshold at 0.35. The dendritiform cells (MRC<0.35) will be further mentioned as Dendritic-shaped Cells (DSCs), (about 16% of the total CX_3_CR1 population), and the round cells (MRC>0.35) will be further mentioned as Round-shaped Cells (RSCs) (about 84% of the total CX_3_CR1 population, including primarily monocytes, but also NK and T cells).

Therefore, subpopulations of DC and monocytes in flow cytometry were in the same range as DSC and RSC in microscopic analysis. We decided to set the MRC threshold to 0.35 for the rest of the analysis.

### LPS differentially increases RSCs and DSCs velocity

Previous studies by two-photon excited fluorescence (TPEF) showed that LPS could activate tracheal DCs [19]. In accordance, in our model, intratracheal injection of LPS induced at the early stage a very significant increase of the velocity of both DSCs and RSCs (p<0.0001), as compared to PBS injection (Figure 5A and 5B). At the late stage we observed an increase of velocity more significant for RSCs (p<0.0001) than for the DSCs (p = 0.0416).

### LPS differentially modifies the confinement of RSCs and DSCs

The Maximal Distance (MD) of DSCs was not affected by LPS (Figure 6A). In contrast, RSC's MD is increased and seems to be stage-dependent, from p = 0.0259 up to p = 0.0018 (Figure 6B). The maximum effect is observed for RSC at the late stage, with a 12% increase of MD after LPS that after PBS delivery (Figure 6C and Figure 6D).

Finally, we compared the Meandering Index (MI) of RSCs and DSCs. Even though LPS increased MD for RSCs, neither of the two populations presented a MI altered by LPS. Furthermore, MI values for all groups were less than 0.4, suggesting that movements observed were not directed by chemotactism [25] (data not shown).

## Discussion

In this study, we show for the first time that we could systematically discriminate two cell populations present in an organ explant and sharing the same staining, using a shape criterion. This may be of paramount importance for the DC subset analysis, as so far no fluorescent protein knock-in mouse strain available is expressed only in a defined DC lineage. To our knowledge the separation of different subsets expressing the same fluorescent tag has been seldomly studied, although it can be a major issue.

In a very recent report on lung exploration by TEPF of CD11c-YFP mouse, Veres *et al.* noted that they could observe two different populations: dendritiform for the DCs, and rounded for presumably macrophages in this particular case [21]. In their study, they could not discriminate those cell behaviors. Our methods improve clearly the time-lapse analysis and could be adapted to very general cases, when cells can be differentiated according to their shape.

In fact, the shape is not a poorly defined character of a cell, as stressed by the fact that DCs were identified and described for the first time only by their shape [26], [27], [28], [29]. The very specific hairy DC shape has been for a very long time the only way to characterize those ‘golden’ cells, before powerful flow cytometer methodology was developed. We propose thus a renewed use for this primordial character.

Thanks to that, we analyze here the effects of LPS instillation by intra-tracheal route on cell motility. Intra-tracheal instillation was chosen in spite of its invasivity (a consequence may be the increase of velocity of DSCs at the early *versus* late stage in Figure 4) in order to improve the reproducibility of injection compared to nasal instillation (data not shown). However, every comparison was performed between PBS and LPS groups in order to focus on the specific effects of LPS, excluding effects of tracheotomy and/or of a rather big volume of liquid injected in the lungs.

LPS is a Gram-negative outer membrane component known to elicit strong immune responses via the Toll-like receptor (TLR) 4 [30]. A previous study, using TLR4^−/−^ adoptive transfer has shown that LPS stimulates indirectly lung DCs through the activation of epithelial cells [19]. Interestingly, lung monocytes and DCs also express TLR4 [31]. We then inferred that DCs and monocytes would respond differentially to a LPS trigger. We have shown here that LPS activation increased more significantly the velocity of RSCs and induced them to go significantly farther. This is consistent with what is known of lung monocyte and DC biology. Lung monocytes are smaller, more mobile cells that respond to an infection alarm. We show here, based on a shape criterion, that RSCs respond very rapidly while DSCs with long, hairy extensions are more prone to move in a very swift manner [5]. The increase of velocity of both populations at the early stage after LPS injection proves that both populations are strongly activated by LPS. The difference between RSCs and DSCs occurs at the late stage after LPS activation, where DSCs move slower than RSCs. This difference of velocity may be due to functional difference between monocytes and DCs: monocytes as circulating cells recruited rapidly on inflammatory sites could benefit from an increased velocity, when DCs including CX_3_CR1 are residing cells involved in sampling task and do not need to migrate faster.

Our results prove the feasibility of the shape-based discrimination of two functionally distinct cell populations, monocytes and DCs, both expressing EGFP via the promoter CX_3_CR1, under LPS mediated inflammatory conditions. The results we present are in accordance with the expected functionality of these two major immune cell populations. DCs movements induced by LPS have been already reported [19]. However, we show that LPS has a stronger effect on RSCs than on DSCs. The shape-based criterion herein described should be taken into account for studying the behaviour in fluorescence microscopy of immune cells subject to infection or inflammatory conditions using transgenic mice expressing the same fluorescent protein in distinct populations.

The new imaging tool we have developed in this study more accurately discriminates DC population from closely related lineages. It could be more universally applied to improve our knowledge of the lung immune system.
